# *Raveneliapiepenbringiae* and *Raveneliahernandezii*, two new rust species on *Senegalia* (Fabaceae, Mimosoideae) from Panama and Costa Rica

**DOI:** 10.3897/mycokeys.41.27694

**Published:** 2018-10-23

**Authors:** M. Ebinghaus, D. Begerow

**Affiliations:** 1 AG Geobotanik, Ruhr-Universität Bochum, Germany Ruhr-University Bochum Bochum Germany

**Keywords:** *Senegalia* rust, rust fungi, Phylogeny, Taxonomy

## Abstract

Two new rust species, *Raveneliapiepenbringiae* and *R.hernandezii* (Pucciniales) on *Senegalia* spp. (Fabaceae) are described from the Neotropics (Panama, Costa Rica). A key to the species on neotropical *Senegalia* spp. is provided. Molecular phylogenetic analyses based on 28S rDNA sequence data suggest that the representatives of *Senegalia* rusts distributed in the neotropics evolved independently from species known from South Africa. This is further supported by the teliospore morphology, which is characterised by uniseriate cysts in the neotropical *Senegalia* rusts and contrasting multiseriate cysts in the paleotropic *Ravenelia* species that infect this host genus.

## Introduction

With more than 200 described species, the genus *Ravenelia* is amongst the most speciose genera within the rust fungi (Pucciniales) (Cummins and Hiratsuka 2003). In the tropics and subtropics, members of this genus parasitise a diverse range of hosts of the legume family (Fabaceae), including Caesalpinioideae, Faboideae and Mimosoideae. Numerous species of *Ravenelia* are known from the neotropics, mostly from Mexico (Cummins 1978), Brazil (Dianese et al. 1993, Rezende and Dianese 2001; Hennen et al. 2005) and Argentina (Hernández and Hennen 2002).

However, in the neotropics, occurrence of *Ravenelia* species is poorly known in other countries such as Panama and Costa Rica. Preliminary checklists of abundant fungi in Central America report only a single species of *Ravenelia* in Panama (*R.entadae*) (Piepenbring 2006) and 18 species of *Ravenelia* in Costa Rica, respectively (Berndt 2004).

Specimens of a rust fungus on *Senegaliahayesii* (Benth.) Britton and Rose were collected in Panama in 2013. Another species of *Ravenelia* was discovered through the analysis of herbarium specimens of the U.S. National Fungal Collections (BPI) on *Senegaliatenuifolia* (L.) Britton and Rose. On the basis of morphological and molecular data, these two specimens were herein analysed and described respectively as *Raveneliapiepenbringiae* and *R.hernandezii*.

## Material and methods

### Light- and electron microscopic investigations

Spores representing different spore stages were scraped from the leaf surfaces of dried herbarium specimens and stained in lactophenol solution on microscope slides. For the analysis of soral structures, hand sections were prepared under a stereomicroscope. Samples were microscopically studied with a Zeiss Axioplan Light Microscope and Zeiss AxioCam. Cellular structures were measured using ZEN 2 (Blue Edition) Software. Infected leaflets of the herbarium specimens were mounted on double-sided sticky carbon tape on metal stubs and coated with gold in a Sputtercoater BAL-TEC SCD OSO (Capovani Brothers Inc, USA). Superficial ornamentation of spores was investigated using a ZEISS Sigma VP scanning electron microscope at the Ruhr-University Bochum, Germany.

### DNA extraction and PCR

Genomic DNA extractions were carried out using the INNUPrep Plant DNA Kit (Analytic Jena, Germany) according to the manufacturer’s protocol. Spores were milled in a Retsch Schwingmühle MM2000 (F. Kurt Retsch GmbH &Co KG, Haan, Germany), using two steel beads and liquid nitrogen in three consecutive cycles. An amount of 40 ml of lysis buffer was added to loosen spore remnants by vortexing from the Eppendorf tube lid, followed by centrifuging in a final cycle. Polymerase chain reaction (PCR) of 28S rDNA was conducted using the Taq-DNA-Polymerase Mix (PeqLab, Erlangen, Germany). To compensate for small amounts of spores applied for DNA extractions up to 5ml of genomic DNA extraction were used as the template in 25 ml reactions. Primer pair LR0R (Moncalvo et al. 1995) and LR6 (Vilgalys and Heester 1990) were used to obtain sequences of the 28S rDNA, with thermal cycling conditions set at 96 °C (3 min) followed by 40 cycles of 30 sec at 95 °C, 40 sec at 49 °C and 1 min at 72 °C, with a final extension for 7 min at 72 °C. PCR products, which showed only weak bands on agarose gels, were purified with Zymo Research DNA Clean & Concentrator-5 Kit (ZymoResearch Corp., Irvine, USA), according to the manufacturer’s protocol. The remaining PCR products were purified using Sephadex G-50 columns (Sigma-Aldrich Chemie GmbH, Taufkirchen, Germany). Sequencing was carried out in both directions using the same primers as in PCR at the sequencing service of the Faculty of Chemistry and Biochemistry of the Ruhr-University Bochum, Germany and by GATC (GATC Biotech, Konstanz, Germany)

### Phylogenetic analyses

Sequences were screened against the NCBI Genbank using the BLAST algorithm to check for erroneously amplified contaminations and were afterwards edited manually using Sequencher 5.0 software (Gene Codes Corp., Michigan, USA). In total, 26 sequences were included (Table 1) to construct an alignment of the 28S rDNA-sequence data using MAFFT v6.832b (Katoh and Standley 2013). Maximum likelihood (ML) analyses were performed with RxML 8.0.26 (Stamatakis 2014) using RAxML GUI v. 1.31 (Silvestro and Michalak 2012) based on the General Time Reversible model of nucleotide substitution plus gamma distribution (GTR+G; Rodriguez et al. 1990) and 1000 generations. Four representative species of *Endoraecium* (KJ862335, KJ862298, KJ862337, KJ862344) were set as multiple outgroups. Maximum Parsimony (MP) analyses were carried out using MEGA6 (Tamura et al. 2013) using the heuristic search option with tree bisection-reconnection (TBR) branch swapping algorithm with 10 initial trees using random step-wise addition. The reliability of topology was tested using the bootstrap method with 1000 replicates.

**Table 1.** Specimens analysed in this study, including GenBank Accession Numbers. Published references are given for sequences obtained from GenBank. †: BPI (U.S. National Fungus Collections, USA); ‡: KR (Staatliches Museum für Naturkunde Karlsruhe, Germany); $: PREM (Plant Protection Research Institute, South Africa); |: Z+ZT (Universität Zürich, Switzerland and Eidgenössische Technische Hochschule Zürich, Switzerland); ¶: BRIP (Department of Agriculture and Fisheries, Australia); #: PMA (Universidad de Panamá, Panama).

Voucher	Species	Substrate	Reference	Origin	LSU
					GenBank
BPI841185†	*Raveneliacohniana* Henn.	*Senegaliapraecox* (Grieseb.) Seigler & Ebinger	This work	Catamarca Province, Argentina	MG954487
BPI841034†	Raveneliaechinatavar.ectypa (Arthur & Holw.) Cummins	*Calliandraformosa* (Kunth) Benth.	Scholler and Aime, 2006	Tucuman Province, Argentina	DQ323925*
KR-M-0043650‡	*Raveneliaescharoides* Syd.	Senegaliaburkei (Benth.) Kyal. & Boatwright	This work	Mpumalanga, South Africa	MG954480
KR-M-0043651‡	*Raveneliaescharoides* Syd.	*Senegaliaburkei* (Benth.) Kyal. & Boatwright	This work	Limpopo, South Africa	MG954481
KR-M-0043652‡	*Raveneliaescharoides* Syd.	*Senegaliaburkei* (Benth.) Kyal. & Boatwright	This work	Limpopo, South Africa	MG954482
PREM61223$	*Raveneliaevansii* Syd.	*Vachelliasieberiana* (Burtt Davy) Kyal. & Boatwr.	This work	KwaZulu-Natal, South Africa	MG945988
PREM61228$	*Raveneliaevansii* Syd.	*Vachelliasieberiana* (Burtt Davy) Kyal. & Boatwr.	This work	KwaZulu-Natal, South Africa	MG945989
PREM61855$	*Raveneliahalsei* Doidge	*Senegaliaataxacantha* (D.C) Kyal. & Boatwright	This work	Mpumalanga, South Africa	MG954484
Z+ZT RB5788|	*Raveneliahavanensis* Arthur	*Enterolobiumcontortisiliquum* (Vell.) Morong	Aime, 2006	Tucuman Province, Argentina	DQ354557*
BPI872308†	*Raveneliahernandezii* Ebinghaus & Begerow	*Senegaliatenuifolia* (L.) Britton & Rose	This work	Guanacaste, Costa Rica	MG954488
PREM61222$	*Raveneliamacowaniana* Pazschke	*Vachelliakarroo* (Hayne) Banfi & Galasso	This work	Limpopo Province, South Africa	MG946007
PREM61210$	*Raveneliamacowaniana* Pazschke	*Vachelliakarroo* (Hayne) Banfi & Galasso	This work	Eastern Cape Province, South Africa	MG946004
PREM61221$	*Raveneliamacowaniana* Pazschke	*Vachelliakarroo* (Hayne) Banfi & Galasso	This work	North-West Province, South Africa	MG946005
BPI841195†	*Raveneliamacrocarpa* Syd. & Syd.	*Sennasubulata* (Griseb.) H.S. Irwin & Barneby	Scholler and Aime 2006	Argentina	DQ323926*
BRIP56908¶	*Ravenelianeocaledoniensis* Huguenin	*Vachelliafarnesiana* (L.) Wight & Arn.	McTaggart et al. 2015	Kununurra, Australia	KJ862348*
BRIP56907¶	*Ravenelianeocaledoniensis* Huguenin	*Vachelliafarnesiana* (L.) Wight & Arn.	McTaggart et al. 2015	Northern Territory, Australia	KJ862347*
KR-M-0045114‡	*Raveneliapienaarii* Doidge	*Senegaliacaffra* (Thunb.) P.J.H. Hurter & Mabb.	This work	Gauteng, South Africa	MG954483
PREM61892$	*Raveneliapienaarii* Doidge	*Senegaliacaffra* (Thunb.) P.J.H. Hurter & Mabb.	This work	KwaZulu-Natal, South Africa	MG954482
MP5157 (PMA)#	*Raveneliapiepenbringiae* Ebinghaus & Begerow	*Senegaliahayesii* (Benth.) Britton & Rose	This work	Chiriquí Province, Panama	MG954489
BRIP56904¶	*Ravenelia* sp.	*Cassia* sp. Mill.	McTaggart et al. 2015	Northern Territory, Australia	KJ862349*
PREM61858$	*Raveneliatransvaalensis* Doidge	*Senegaliamellifera* (Vahl) Seibler & Ebinger	This work	North-West Province, South Africa	MG954485
PREM61893$	*Raveneliatransvaalensis* Doidge	*Senegaliamellifera* (Vahl) Seibler & Ebinger	This work	North-West Province, South Africa	MG954486
BRIP56539¶	*Endoraeciumauriculiforme* McTaggart & Shivas	*Acaciadifficilis* Maiden	McTaggart et al., 2015	Northern Territory, Australia	KJ862398*
BRIP27071¶	*Endoraeciumtierneyi* (Walker & Shivas) Scholler & Aime	*Acaciaharpophylla* F.Muell. ex Benth.	McTaggart et al. 2015	Queensland, Australia	KJ862335*
BRIP56557¶	*Endoraeciumtropicum* McTaggart & Shivas	*Acaciatropica* (Maiden & Blakely) Tindale	McTaggart et al. 2015	Northern Territory, Australia	KJ862337*
BRIP56545¶	*Endoraeciumviolae-faustiae* Berndt	*Acaciadifficilis* Maiden	McTaggart et al. 2015	Northern Territory, Australia	KJ862344*

## Results

### Phylogenetic analyses

**Figure 1.**
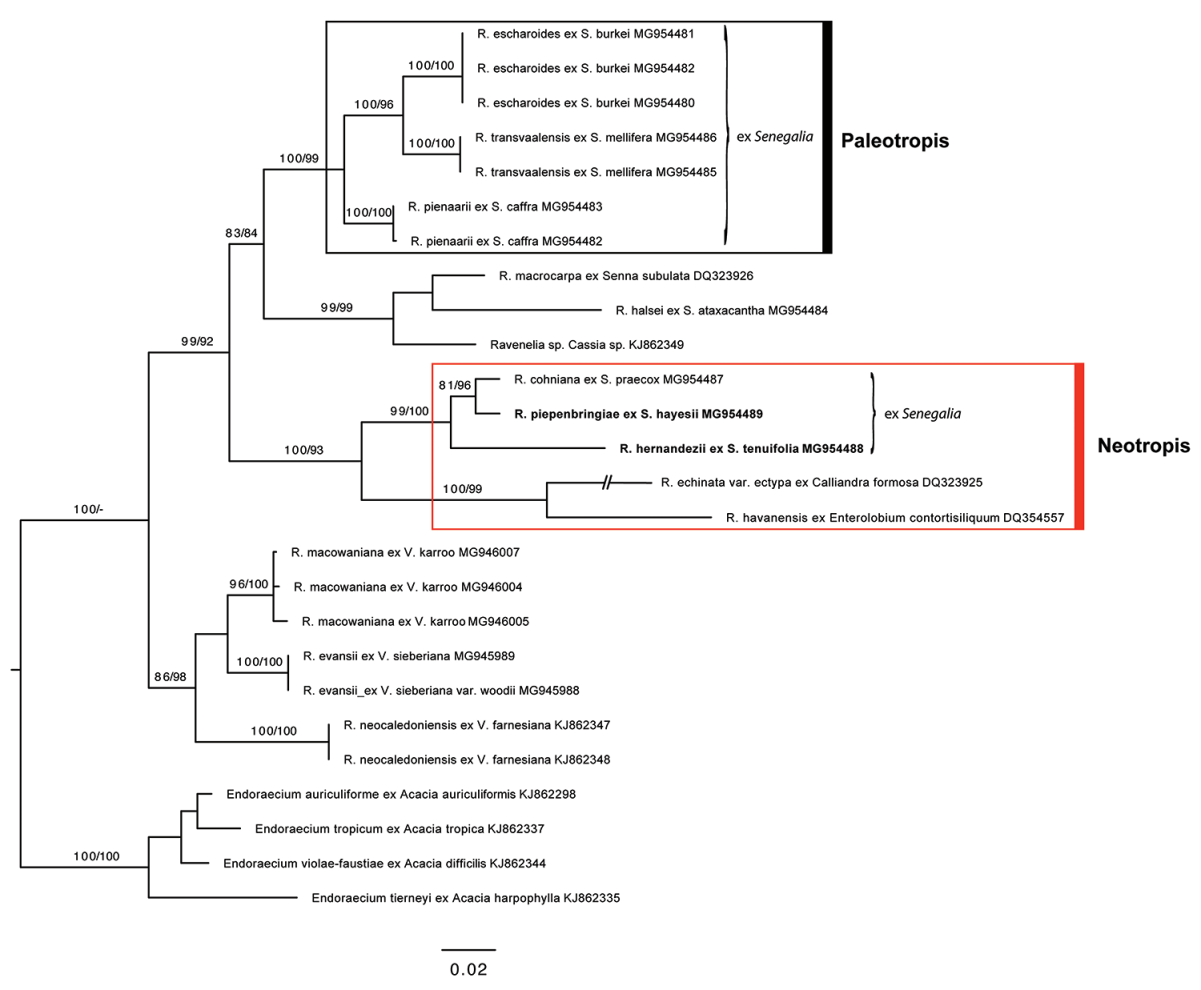
Maximum likelihood reconstruction of *Ravenelia* spp. based on 28S rDNA sequence data. Bootstrap values are shown above branches based on 1000 replicates (MLBS and MPBS, respectively), values below 75 are not shown. Names of species collected on neotropical *Senegalia* hosts including *R.piepenbringiae* and *R.hernandezii* are highlighted (bold, red box). For paleotropically distributed species of *Senegalia* rusts, see black box.

The alignment of the 28S rDNA sequence data consisted of 26 sequences representing 18 taxa and had a total length of 1015 nucleotides with 305 variable characters, 250 parsimony-informative sites and 55 singletons. The tree topologies of MP and ML analyses were identical and thus only the ML tree is shown. A clade, comprising rusts on neotropical *Senegalia* species, i.e. *R.cohniana*, *R.hernandezii* sp. nov. and *R.piepenbringiae* sp. nov., displays a robustly supported sister-group (MLBS/MPBS = 99/100) to two neotropically distributed rusts which infect non-*Senegalia* hosts (i.e. R.echinatavar.ectypa on *Calliandraformosa*, DQ323925 and *R.havanensis* on *Enterolobiumcontortisiliquum*DQ354557) (Scholler and Aime 2006, Aime 2006). A second clade, based on sequences obtained from *Ravenelia* species on *Senegalia* spp. with paleotropical origin, appeared only distantly related to the former species cluster (MLBS/MPBS = 100/99) (Figure 1).

### Taxonomy

#### Ravenelia piepenbringiae

Taxon classificationFungiPuccinialesRaveneliaceae

Ebinghaus & Begerow, sp. nov. on Senegalia hayesii (Benth.) Britton & Rose (Mimosoideae, Leguminosae)

Mycobank: MB 824297

Fig. 2

##### Type.

Panama, Chiriquí Province, Dolega District, Los Algarrobos, Casa de la Alemana, Bosquecito, approx. 150 m a.s.l., 8°29'45.31"N, 82°25'56.24"W on *Senegaliahayesii* (Benth.) Britton and Rose, 17 February 2013, coll. M. Piepenbring MP 5157 [**holotype**: s.n. (PMA), isotypes: KR-M-0043654 (KR). M-0141345 (M)]

##### Etymology.

Named after M. Piepenbring, who discovered the rust fungus in her garden and provided the specimens.

Spermogonia and aecia not seen. Uredinia hypophyllous, single or in irregular groups, light brown, often associated with necrotic spots that are also evident on the adaxial surface, 0.1–0.8 mm in diameter, aparaphysate, subepidermal, covered by the epidermis when young, later erumpent. Urediniospores obovoidal, ellipsoidal or slightly curved, often limoniform with an acuminate apex, ochraceous brown, (18)21–25(29) × 12–15(20) mm; spore wall laterally 1–1.5 mm thick, apically and basally often slightly thickened, distinctly verrucose to echinulate; aculei 1.0–1.5 mm high, distances between aculei about 2 mm, germ pores 4–7, in equatorial position. Telia replacing uredinia or developing independently from uredinia, chestnut to dark brown, sometimes confluent. Teliospores roundish to broadly ellipsoidal to oblong in planar view, hemispherical in lateral view, with 4–6 probasidial cells across, single-layered, each teliospore formed by 9–13 probasidial cells, (44)58–73(78) mm in diameter, single probasidial cells (19)22–26(31) × (11)17–22(28) mm; cell wall thickened at the surface of the teliospore (epispore), 2–4(5) mm thick, often with a thin and hyaline outer layer, each probasidial cell with 7–11 rod-shaped, straight spines that are (1)2–3(4.5) mm long; cysts at the basis of the teliospores, uniseriate and in the same position and number as the peripheral probasidial cells, globose, hyaline, swelling in water, slightly swelling in lactophenol.

Further specimens. Type locality, 22 January 2014, M. Piepenbring 5203 [M-0141344 (M), s.n. (UCH)]. Type locality, 12 January 2017, M. Piepenbring & I. D. Quiroz González 5333 (UCH, s.n.).

**Figure 2.**
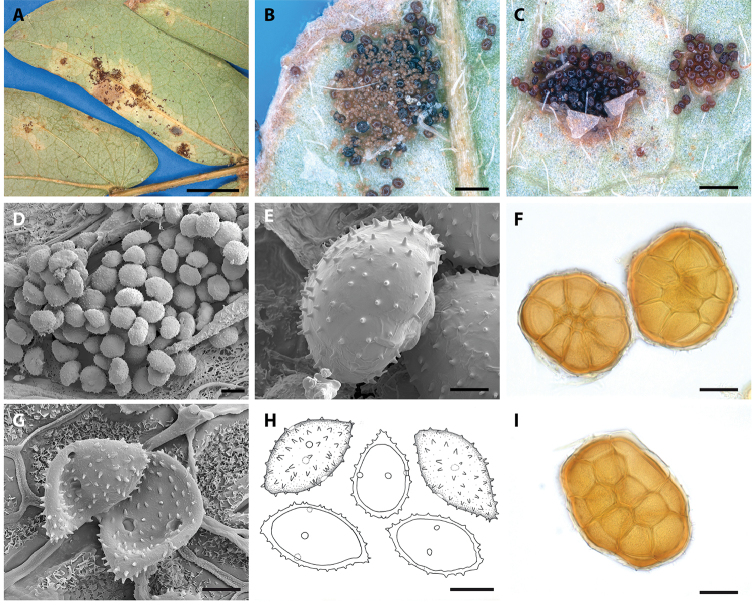
*Raveneliapiepenbringiae*. **A** Telia in chlorotic spots associated with infection of *Senegaliahayesii***B, C** sori showing uredinio- and teliospores and teliospores, respectively **D** SEM image of a telium **E** SEM view of a teliospore **F, I** LM images of teliospores **G** SEM image of urediniospores showing equatorially arranged germ pores **H** drawings of urediniospores. Scale bars: 3 mm (**A**); 0.1 mm (**B**); 0.2 mm (**C**); 40 mm(**D**); 10 mm (**E**); 20 mm(**F**); 5 mm(**G**); 10 mm(**H**); 20 mm(**I**).

#### Ravenelia hernandezii

Taxon classificationFungiPuccinialesRaveneliaceae

Ebinghaus & Begerow, sp. nov. on Senegalia tenuifolia (L.) Britton and Rose (Mimosoideae, Leguminosae)

Mycobank: MB 824298

Fig. 3

##### Type.

Costa Rica, Guanacaste, Area de Conservación Guanacaste, Sendero Bosque húmedo (10°50.702'N, 85°36.450'W) on *Senegaliatenuifolia* (L.) Britton and Rose, coll. J.R. Hernandez, 1. December 2003. Holotype: BPI 872308 (BPI).

##### Etymology.

Named after J.R. Hernández who collected the type specimen.

Spermogonia and aecia not seen. Uredinia hypophyllous, minute, single or in small and often loose groups, ochraceous to light brown, 0.1–0.3 mm in diameter, aparaphysate, subepidermal, erumpent and surrounded by torn epidermis; urediniospores obovoidal, ellipsoidal, often reniform or slightly curved, ochraceous brown, often with an attached fragment of the pedicel, (17)18–21(24) × (8)9–10(12) mm; spore wall thin, laterally (0.5)1–1.5 mm thick, apically and basally slightly thickened, distinctly echinulate; aculei approximately 1.0–1.5 mm high, germ pores 5–6, in equatorial position. Telia replacing uredinia, chestnut- to dark brown. Teliospores (59)67–75(96) mm, roundish or broadly ellipsoidal to oblong in planar view, hemispherical in lateral view, 5–6 probasidial cells across, single-layered, central cells often arranged in two rows of 3 or 4 cells, each cell (19)22–25(39) × (11)17–22(28) mm, cell wall thickened at the apex, (2.5)3.0–4.5(6.0) mm thick, often with a thin and hyaline outer layer, probasidial cells each with 3–5 rod-shaped straight spines (1)3–4(6) mm long; cysts on the abaxial side of the teliospores, uniseriate and in same position and number as the peripheral probasidial cells, globose, hyaline, swelling in water, slight swelling in lactophenol.

**Figure 3.**
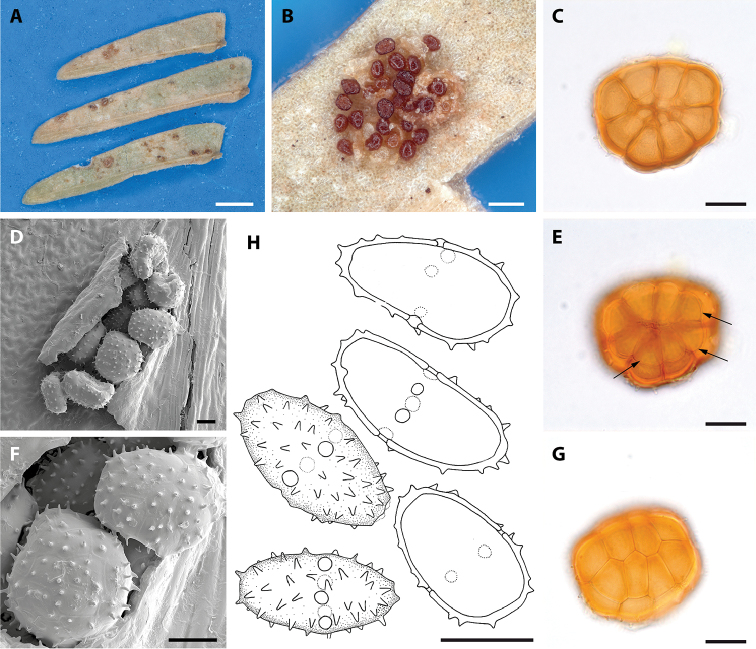
*Raveneliahernandezii*. **A** Infected leaflets of *S.tenuifolia***B** Mixed sori containing urediniospores and teliospores **C** Teliospore seen in LM **D** telium seen by SEM **E** Adaxial view of a teliospore by LM, with arrows indicating the uniseriate cysts **F** SEM view of spinescent teliospores **G** LM view of the upper surface **H** drawing of a urediniospore. Scale Bars: 0.5 mm (**A**); 0.1 mm (**B**); 20 mm (**C–G**); 10 mm (**H**).

## Discussion

A total of 10 species of *Ravenelia* have been described to date from the neotropics parasitising *Senegalia* trees: *R.cohniana* Hennings on *S.praecox* (Griseb.) Seigler & Ebinger, *R.idonea* Jackson & Holway, *R.lata* Hennen & Cummins on *S.glomerosa* (Benth.) Britton & Rose, *R.monosticha* Speg. on *S.bonariensis* (Gillies ex Hook. & Arn.) Seigler & Ebinger, *R.pringlei* Cummins on *S.greggii* (A. Gray) Britton & Rose, *R.rata* Jackson & Holway on *S.pedicellata* (Benth.) Seigler & Ebinger, *R.roemerianae* Long on *S.roemeriana* (Scheele) Britton & Rose, *R.scopulata* Cummins & Baxter on *S.greggii* (A. Gray) Britton & Rose, *R.stevensii* Arthur on *S.riparia* (Kunth) Britton & Rose ex Britton & Killip and *R.versatilis* (Peck) Dietel on *S.anisophylla* (Watson) Britton & Rose. No species of *Ravenelia* has been reported to affect *Senegaliahayesii* or *S.tenuifolia*. Most of these species known to parasitise *Senegalia* spp. are distinguished from species identified in this study by abundant paraphyses in the uredinia, except for *Raveneliarata* which also lacks paraphyses in the uredinia. However, this species differs from *R.piepenbringiae* and *R.hernandezii* by abundant tuberculate teliospore ornamentations 2–3µm in length and by formation of only 2–4 cysts per teliospore. Both newly described species exhibit longer tuberculate spines and bear 6–8 cysts per teliospore. *Raveneliacohniana* is the only species that resembles various teliospore and urediniospore characteristics of *R.piepenbringiae* and *R.hernandezii* (see Table 2). The teliospores of *R.hernandezii*, however, are larger in size than those of the latter two species (Table 2). In contrast to the teliospores, urediniospores of *R.hernandezii* tend to be smaller and more slender, while they mostly lack the characteristic acuminate apex present in urediniospores of *R.piepenbringiae* (Table 2; compare Figures 1H and 2H). Hernández and Hennen (2002) considered *R.concinna* Syd. on *S.riparia* (Kunth) Britton & Rose ex Britton & Killip and *S.glomerosa*, *R.distans* Arthur & Holway on an unidentified mimosoid host and *R.lindquistii* Hennen & Cummins on *Senegaliapraecox* as synonyms of *R.cohniana* due to a nearly identical morphology. However, given the likewise close morphological resemblance in *R.piepenbringiae*, *R.hernandezii* and *R.cohniana*, despite being phylogenetic entities, this assumption needs revision by molecular phylogenetic means.

The resemblance of teliospore characters in *R.cohniana* and the species identified in the present study suggests a close relationship which is supported by the phylogenetic reconstructions. These neotropical rusts on *Senegalia* further appear to have evolved independently from those *Senegalia* rusts that have a paleotropic origin (Fig. 1, Table 1). The phylogenetic distinction of both lineages is also mirrored by a morphological feature: the arrangement of teliosporic cysts is uniseriate in the analysed neotropic species but multiseriate in all investigated paleotropic *Senegalia* rusts (Table 2).

**Table 2.** Summary of morphological characteristics of *Ravenelia* species infecting *Senegalia* trees in the neotropics. All measurements are given in mm. Absent characters are indicated with dashes.

**Species**	**Teliospore characters**											**Source**
	**Teliospore size**		**Probasidial cell size**		**Epispore**		**Ornamentation**			**Cells in Diameter**	**Arrangement of Cysts**	
							**Number per cell**	**length**	**shape**			
* R. cohniana *	(39)45–73(74)		16–22 × 13–15		not stated		(2)3–5(8).	3–5	spinescent	(3)4–5(6)	uniseriate	Hernández and Hennen (2002)
* R. escharoides *	55–90		30–35 × 16–20		up to 6		4–9	1–2	verrucose	6–8	multiseriate	Doidge (1939)
* R. halsei *	80–112		25–30 × 10–15		5–6		–	–	smooth	9–11	uniseriate	Doidge (1939)
* R. hernandezii *	(59)67–75(96)		(19)22–25(39) × (11)17–22(28)		(2.5)3–4.5(6)		3–5	(1)3–4(6)	spinescent	5–6	uniseriate	This study
* R. lata *	53–64		(18)22–26 (width)		not stated		6–20	not stated	spinescent	4	multiseriate	Hennen et al. (2005)
* R. monosticha *	(50)53–55 × 65–70		16–19 × 13–15		not stated		4–8	not stated	verrucose	4–6	uniseriate	Spegazzini (1923)
* R. pienaarii *	80–120		25–30 × 10–15		up to 7		4–7	1–1.5(2)	verrucose	(6)7–10	multiseriate	Doidge (1939)
* R. piepenbringiae *	(44)58–73(78)		(19)22–26(31) × (11)17–22(28)		2–4(5)		7–11	(1)2–3(4.5)	spinescent	4–6	uniseriate	This study
* R. pringlei *	(55)70–95(105)		(12)14–18(20) (width)		not stated		not stated	not stated	verrucose	(5)6–8	uniseriate	Cummins (1975)
* R. rata *	(30)33–40(44)		14–20 × 12–17		1.5		not stated	2–3	verrucose	2–4	uniseriate	Hennen et al. (2005)
* R. roemerianae *	63–100		Not stated		not stated		3–10	2	verrucose	5–7	uniseriate	Long (1917)
* R. scopulata *	(55)65–100(110)		(13)16–19(21) (width)		not stated		not stated	not stated	smooth	5–8	multiseriate	Cummins and Baxter (1976)
* R. stevensii *	40–63		Not stated		not stated		1–3	6–19	verrucose	3–6	multiseriate	Arthur (1915)
* R. transvaalensis *	75–100		30–35 × 15–17.5		up to 6		–	–	smooth	5–6	multiseriate	Doidge (1939)
* R. versatilis *	85–105		10–16 (width)		not stated		–	–	smooth	7–9	not stated	Dietel (1894)
	**Paraphyses**				**Urediniospore characters**							**Source**
	Position	Shape		Size		Cell wall		Germ pores		Shape		
								Number	Position			
* R. cohniana *	–	–		(12)20–28(32) × (11)13–17(19)		1.5–2.5(3)		(3)4(6)	equatorial	oblong-ellipsoidal		Hernández and Hennen (2002)
* R. escharoides *	–	–		17–22×14–17		1.5		Not stated	not stated	obovoidal-ellipsoidal		Doidge (1939)
* R. halsei *	not stated	not stated		–		–		–	–	–		Doidge (1939)
* R. hernandezii *	–	–		(17)18–21(24) × (8)9–10(12)		(0.5)1–1.5		5–6	equatorial	obovoidal-ellipsoidal		This study
* R. lata *	peripheral	capitate		(22)25–32(36) × (12)14–17(18)		1.5–2		(4)5–6	equatorial	obovoidal-oblong		Hennen et al. (2005)
* R. monosticha *	peripheral	capitate		(23)26–30(33) × (8)12–14(15)		1.5–2		4–5(6)	equatorial	obovoidal-ellipsoidal		Spegazzini (1923)
* R. pienaarii *	–	–		20–25 × 15–19		1.5		6	equatorial	ellipsoidal-subglobose		Doidge (1939)
* R. piepenbringiae *	–	–		(18)21–25(29) × 12–15(20)		1–1.5		4–7	equatorial	obovoidal-limoniform		This study
* R. pringlei *	not stated	clavate - capitate		(10)11–15(17) × (20)26–33(35)		(1)1.5(2)		8	bizonate	oblong-ellipsoidal		Cummins (1975)
* R. rata *	–	–		–		–		–	–	–		Hennen et al. (2005)
* R. roemerianae *	intrasoral	clavate		10–14 × 27–38		1–1.5		8	bizonate	obovoidal-oblong		Long (1917)
* R. scopulata *	not stated	clavate		(17)19–24 × (11)12–14(15)		(1)1.5(2)		6–8	bizonate	oblong-ellipsoidal		Cummins and Baxter (1976)
* R. stevensii *	peripheral	clavate - capitate		8–13 × 25–30		<1		4	equatorial	oblong-obovoidal		Arthur (1915)
* R. transvaalensis *	–	–		–		–		–	–	–		Doidge (1939)
* R. versatilis *	intrasoral	clavate - capitate		13–18 × 26–32		Not stated		8	bizonate	obovoidal-oblong		Dietel (1894)

### Key to species of Ravenelia infecting neotropical Senegalia trees

**Table:** 

1	Teliospores ≤64 mm; urediniospores with equatorially arranged germ pores	**2**
–	Teliospores >64 mm; urediniospores with bizonate or equatorially arranged germ pores	**4**
2	Paraphyses present in uredinia	**3**
–	Paraphyses absent in uredinia	*** R. rata ***
3	Teliospores with <6 verrucae per cell; on *S.riparia*	*** R. stevensii ***
–	Teliospores with 6–20 spines per cell; on *S.glomerosa*	*** R. lata ***
4	Urediniospores with 6–8 bizonate germ pores; teliospores verrucose or smooth	**5**
–	Urediniospores if present with equatorially arranged germ pores; teliosporesspinescent or verrucose; teliospore cysts uniseriate	**8**
5	Teliospores smooth	**6**
–	Teliospores verrucose	**7**
6	On *S.anisophylla*; urediniospores 12–14 × 19–24 mm	*** R. versatilis ***
–	On *S.greggii*; urediniospores 13–18 × 26–32 mm	*** R. scopulata ***
7	With intrasoral paraphyses; on *S.roemeriana*	*** R. roemerianae ***
–	On *S.greggii*	*** R. pringlei ***
8	Paraphyses present; teliospores verrucose; on *S.bonariensis*	*** R. monosticha ***
–	Paraphyses absent; teliospores spinescent	**9**
9	Teliospores with 7–11 spines per cell; urediniospores often limoniform; on *S.hayesii*	*** R. piepenbringiae ***
–	Teliospores with 3–5 spines per cell; urediniospores obovoidal to ellipsoidal,sometimes limoniform	**10**
10	Teliospores 59–96 mm in diameter; urediniospores <13mm in width; urediniospore wall laterally 1–1.5 mm; on *S.tenuifolia*	*** R. hernandezii ***
–	Teliospores 39–75 mm in diameter; urediniospores 11–19 mm in width; urediniospore wall laterally 1.5–2.5 mm; on *S.praecox*	*** R. cohniana ***

## Supplementary Material

XML Treatment for Ravenelia piepenbringiae

XML Treatment for Ravenelia hernandezii
